# Preventive Pharmacovigilance: timely and precise prevention of adverse events through person-level patient screening and dose-level product surveillance

**DOI:** 10.1007/s11095-023-03548-3

**Published:** 2023-06-22

**Authors:** Yihua Bruce Yu, Katharine T. Briggs, Marc B. Taraban

**Affiliations:** 1 Bio and Nano-technology Center, University of Maryland School of Pharmacy, 20 North Penn Street, Baltimore, MD 21201 USA; 2 Institute for Bioscience and Biotechnology Research, 9600 Gudelsky Drive, Rockville, MD 20850 USA

**Keywords:** dose-level product surveillance, measurement science, person-level patient screening, preventive pharmacovigilance, vaccine safety

## Abstract

The goal of pharmacovigilance (PV) is to prevent adverse events (AEs) associated with drugs and vaccines. Current PV programs are of a reactive nature and rest entirely on data science, i.e., detecting and analyzing AE data from provider/patient reports, health records and even social media. The ensuing preventive actions are too late for people who have experienced AEs and often overly broad, as responses include entire product withdrawals, batch recalls, or contraindications of subpopulations. To prevent AEs in a timely and precise manner, it is necessary to go beyond data science and incorporate measurement science into PV efforts through person-level patient screening and dose-level product surveillance. Measurement-based PV may be called ‘preventive pharmacovigilance’, the goal of which is to identify susceptible individuals and defective doses to prevent AEs. A comprehensive PV program should contain both reactive and preventive components by integrating data science and measurement science.

Vaccination on the global scale during the COVID-19 pandemic brought vaccine safety and adverse events to the fore of public discourse. It has become increasingly clear that preventing vaccine-associated adverse events (AEs) is crucial to the success of vaccination programs. In fact, the need to prevent AEs extends far beyond vaccines. The World Health Organization (WHO) defines pharmacovigilance (PV) as “the science and activities related to the detection, assessment, understanding and prevention of adverse drug effects or any other possible drug-related problems.” [1].

The WHO describes PV as safety monitoring [1] while the FDA describes PV as safety surveillance [2]. Current PV efforts focus on detecting and analyzing safety signals, i.e., AEs, either passively, through programs such as the VAERS and FAERS programs in the US, which rely on healthcare provider reporting or patient self-reporting after an AE, or actively, such as through the Sentinel Initiative in the US, which gathers data from medical claims and electronic health records, [3]. In the European Union, the EMA operates the EudraVigilance Program for managing and analyzing AE reports. Globally, the WHO has PV programs such as the Programme for International Drug Monitoring (PIDM) and the Vaccine Safety Net (VSN), which focus on collecting and sharing drug and vaccine safety signals.

Although the WHO’s definition of PV includes prevention, current PV efforts are of a *reactive* nature, i.e., reacting to adverse events (AEs) that have already happened. Based on the safety signals, regulatory agencies such as the FDA, the EMA and health authorities, such as the WHO, may issue warnings, update product labeling (e.g., adding contraindications), initiate recalls of specific batches/lots, and reevaluate the risk-benefit balance and approval decisions. Although product warnings, label updates and approval reevaluations may help prevent future AEs, they are made only after a significant number of serious AEs have been reported or detected. For those who have experienced AEs, AE prevention by reactive PV efforts is *too late*.

AE prevention by reactive PV is not only late, but often *imprecise*, too, thus necessitating an overreaction to remedy the problem. Preventive actions following AE detection may include the withdrawal of an entire product, the recall of an entire batch, or contraindication for an entire subpopulation. For example, Omontys^®^ was withdrawn less than one year after its regulatory approval due to reports of anaphylaxis and death among patients receiving the drug, even though only one of the two presentations of this drug was associated with the AEs [4]. In 2013, thirty-three batches of Novomix-30^®^ were recalled even though only 0.14% of the insulin pens in these batches contained the wrong insulin concentration [5]. A more recent example is the recall of three batches of the Moderna COVID-19 vaccine in Japan after particulates were found in a tiny fraction of the vials in those batches [6, 7]. As for contraindications, whether mRNA COVID-19 vaccines, which contain polyethylene glycol (PEG), should be contraindicated for people with past allergies to PEG has been a matter of debate/confusion [8]. The root cause of preventive overreaction is that AE data usually cannot pinpoint which individuals are predisposed to develop AEs to a product, hence an entire subpopulation is contraindicated, or which individual doses are defective to cause harm, hence entire products are withdrawn or entire batches are recalled.

In general, AEs may be caused by patient factors (e.g., allergic to an ingredient of the product), product factors (e.g., defective or falsified products), or both. An example of AEs involving both patient and product factors is the epoetin analogue HX575, the clinical trial of which at 89 medical centers in Asia and Europe was terminated in 2009 after two of the 174 participants developed series AEs. Eight years and multiple studies later, the likely cause of the AEs was revealed to be a combination of patient factors (certain genetic alleles present in some but not all of the patients) and product factors (high level of protein aggregates in some but not all of the prefilled syringes) [9].

During the COVID-19 pandemic, quarantines of entire populations, a preventive overreaction based on experience with past pandemics (historical data), was gradually fine-tuned to quarantines of individuals who tested positive for COVID-19, which was made possible by real-time, on-site measurements that pinpointed infected individuals. In the realm of drug safety, skin tests of individual patients before injection can prevent AEs caused by some drugs, such as penicillin, in a timely and precise manner. Without those skin tests (real-time, on-site measurements), penicillin might have been withdrawn from the market after the occurrence of significant serious AEs.

From a technical standpoint, current PV is based exclusively on data science, i.e., mining and analyzing AE data of various types from different sources [10]. As such, its preventive power is limited by the predictive power of historical data (e.g., people allergic to product X that contains ingredient Y may be allergic to product Z that also contains ingredient Y). In contrast, other types of preventive actions, such as weather, air quality, and pollen forecasting, or earthquake, tsunami, and tornado warnings, all rely on a combination of both data science (analyzing data of past events to look for patterns) and measurement science (collecting and analyzing data on current events). In the public health arena, the prevention and containment of a pandemic also relies on both data science (lessons learned from past pandemics) and measurement science (real-time, on-site testing).

Preventive overreactions based on historical AE data amount to collective punishment with broad negative impacts. Withdrawing a product or terminating a clinical trial because of a few cases of serious AEs may deprive many patients of safe and efficacious products. The reason is that the few serious AEs may be the result of a few overly sensitive individuals and/or a few defective doses, as illustrated by the case of the epoetin analogue HX575. Recalls of entire batches may cause drug/vaccine wastage and even shortage, and contraindication of a subpopulation may unnecessarily restrict patient access. Preventive overreactions may also inflict severe costs, financial and otherwise, on industry and the broader healthcare system.

To prevent AEs in a more timely and precise manner, we suggest incorporating real-time, on-site measurements into the PV landscape. Measurement-based PV can be called preventive PV. Considering that AEs may be caused by both patient and product factors, preventive PV should include quantitative measurements on both patients and products, with the long-term goal of quantitatively testing every person (person-level patient screening) and every dose (dose-level product surveillance) before usage. The goal is to combine person-level patient screening with dose-level product surveillance to prevent most AEs from happening, wherever possible. The actual practice would likely be nuanced and depend on the product type and risk assessment.

The need to conduct patient screening at the person level rather than at the (sub)population level is perhaps obvious; people have different biology (genetics, preexisting conditions, etc.). Indeed, testing individual patients has been practiced for some time, from skin tests to genomic tests. Through a culmination of data and measurement science efforts, it becomes increasingly clear that a drug or vaccine may be safe and effective for many, but not all people. The challenge here is two-fold: to develop fast, affordable and robust patient screening tests for various drugs and vaccines (a challenge for measurement science) and to incorporate patient screening results into the PV data infrastructure (a challenge for data science).

The need to conduct product surveillance at the dose level rather than the batch level might be less obvious. One might think that all doses of a product in a batch are the same or sufficiently similar, provided they are manufactured by a reputable firm using state-of-the-art technologies. But this may not be the case. Pharmaceutical manufacturing, in general, is not very precise [11] and manufacturing of biologics, such as vaccines, is highly complex. Manufacturing errors could result in defective doses that may escape batch-level quality control. Biologics, in general, are fragile and often require stringent handling procedures. Mishandling during distribution, such as cold chain breaches and excessive shaking, may result in additional defective doses. Manufacturing errors and distribution mishandling may render some doses in a batch to be ineffective or even harmful at the point of care. Further, different batches may have uneven quality, depending on their production and distribution history. Falsification and shoddy manufacturing practices exacerbate the issue of product quality. In essence, all doses are not the same when they are administered to people [12]. A recent study reported batch-dependent safety of an mRNA COVID-19 vaccine in the Danish population [13]. It remains to be clarified whether the observed batch-dependent product safety is caused solely by patient factors (e.g., some batches were given to more sensitive people), product factors (e.g., some batches contain more defective doses), or a combination of both. But without data on individual doses, such clarification is challenging.

In 2021, the FDA received 16,105 drug product quality defect reports [14]. As drugs and vaccines are made and distributed globally, the occurrence of poor-quality products may arise, especially in low-resource locales, where distribution and testing facilities may be lacking, and regulatory oversight may be limited. This would make the occurrence of poor-quality products more likely, but their detection less likely in low-resource locales.

Manufacturing errors, substandard or dated manufacturing practices, distribution mishandling and falsification may happen anywhere in the pharmaceutical supply chain, from the point of production to the point of care. Accordingly, dose-level product surveillance may be necessary throughout the supply chain, including in remote and low-resource locales to be conducted by people working in the field (local health authorities, distributors, providers, etc.).

Point-of-care (POC) testing for disease diagnosis is already in practice [15]. Preventive PV, in essence, expands POC testing from diagnosis to therapy and vaccination by combining the testing of individual patients and individual doses to minimize adverse reactions to drugs and vaccines.

Conducting person-level patient screening and dose-level product surveillance at scale is impractical currently, both technologically and financially. New enabling technologies are needed. Person-level patient screening and dose-level product screening technologies need to be quick, quantitative, affordable, robust, and easy to implement with minimal infrastructure and human expertise required. Dose-level product surveillance technologies also need to be noninvasive so that the product may still be administered to patients if deemed of good quality. The integration of person-level patient screening and dose-level product surveillance would require significant advances in both measurement science and data science and corresponding regulatory guidelines.

The myriad and daunting challenges notwithstanding, measurement-based preventive PV is a worthy pursuit. Its true value goes far beyond preventing individual AEs. Measurement-based PV can make preventive actions more precise by pinpointing sensitive individuals and/or defective doses, thereby avoiding overreactions. For example, when integrated into clinical trials, preventive PV may save some promising drug candidates. The aforementioned epoetin analogue HX575 is an example. Had the prefilled syringes with high levels of protein aggregates been detected and removed before injection, the clinical trial of HX575 might have been saved, in addition to having the individual AEs prevented.

Preventive PV may also save some approved products from unnecessary withdrawals. The aforementioned Omontys^®^ is an example. A study conducted by FDA scientists, after the product withdrawal, observed a much higher level of subvisible particles in the multiuse vials (MUVs) than in the single-use vials (SUVs) of the product [4]. The clinical trial of Omontys^®^ primarily used SUVs but it was the MUVs that were marketed. Had the high level of subvisible particles in MUVs been detected before usage, the withdrawal of Omontys^®^ might have been avoided, in addition to having the individual AEs prevented.

Data collected by preventive PV may help pharmaceutical manufacturers, distributors and healthcare providers to better identify deficiencies in their operations. The data may also facilitate regulatory oversights.

Perhaps the biggest value of preventive PV lies in its potential to enhance and restore public trust in medications in general, and vaccines in particular [16]. Once AEs happen, their impact may go far beyond the individuals directly affected the AEs. One example is that in 2014 in Italy, three elderly people died within 48 hours after taking the flu vaccine Fluad^®^. To our knowledge, the cause of the deaths is unclear to this date. Generally, establishing a causal link between AEs and drugs/vaccines is very difficult and time-consuming. However, as a result of extensive media coverage, the vaccination rate in Italy dropped by 12% in 2014 and, coincidentally, the mortality rate in Italy in 2015 increased by 9.1% [17]. Statements made by health authorities after a quick investigation like ‘no evidence of causal link between AEs and vaccination has been identified’ may have limited persuasive power in convincing the public that a vaccine is safe. Preventing vaccine-associated AEs in the first place is likely to be more effective in combating vaccine hesitancy. In essence, preventive PV should be an integral part of preventive medicine.

Preventive PV, rooted in measurement science, will not replace reactive PV, rooted in data science. Preventive and reactive PV can and should work together and in parallel to minimize AEs. The starting point is to recognize that many AEs could be prevented. By preventing AEs in the first place, preventive PV reduces the workload of reactive PV, akin to vaccinations reducing the workload of infectious disease care. The transborder nature of the pharmaceutical supply chain, the danger of world-wide pandemics and the need for global health equity constitute an urgent call to action on preventive pharmacovigilance through technology development.

Quantitative person- and dose-level data may fundamentally transform the risk-benefit analysis for pharmaceutical products, reduce the failure rate of clinical trials, increase patient access to life-saving drugs and vaccines, and cut healthcare costs.
