# Correction: Preventive Pharmacovigilance: timely and precise prevention of adverse events through person-level patient screening and dose-level product surveillance

**DOI:** 10.1007/s11095-023-03575-0

**Published:** 2023-08-10

**Authors:** Yihua Bruce Yu, Katharine T. Briggs, Marc B. Taraban

**Affiliations:** 1 Bio and Nano-Technology Center, University of Maryland School of Pharmacy, 20 North Penn Street, Baltimore, MD 21201 USA; 2 Institute for Bioscience and Biotechnology Research, 9600 Gudelsky Drive, Rockville, MD 20850 USA

**Correction: Pharmaceutical Research**

https://doi.org/10.1007/s11095-023-03548-3

The article Preventive Pharmacovigilance: timely and precise prevention of adverse events through person-level patient screening and dose-level product surveillance, written by Yihua Bruce Yu, Katharine T. Briggs and Marc B. Taraban, was originally published electronically on the publisher’s internet portal on 22-June-2023 without open access. With the author(s)’ decision to opt for Open Choice the copyright of the article changed on 20-July-2023 to © The Authors 2023 and the article is forthwith distributed under a Creative Commons Attribution 4.0 International License, which permits use, sharing, adaptation, distribution and reproduction in any medium or format, as long as you give appropriate credit to the original author(s) and the source, provide a link to the Creative Commons licence, and indicate if changes were made. The images or other third party material in this article are included in the article’s Creative Commons licence, unless indicated otherwise in a credit line to the material. If material is not included in the article’s Creative Commons licence and your intended use is not permitted by statutory regulation or exceeds the permitted use, you will need to obtain permission directly from the copyright holder.

